# Association between exposure to indoor PM_2.5_ constituents and mortality risk among chinese older adults: a nationwide prospective cohort study

**DOI:** 10.1186/s12889-026-29130-1

**Published:** 2026-09-07

**Authors:** Ya Wen, Wan Ying Liu, Jin Wang, Ruixia Niu, Guiming Zhu, Qian Gao, Tong Wang

**Affiliations:** 1 Department of Health Statistics, School of Public Health, Shanxi Medical University, No. 98, University Street, Yuci District, Jinzhong, Shanxi 030600 China; 2 Key Laboratory of Coal Environmental Pathogenicity and Prevention, Shanxi Medical University, Ministry of Education, Taiyuan, 030001 China

**Keywords:** Indoor air pollution, PM_2.5_, Chemical components, Mortality risk, Older adults

## Abstract

**Background:**

With global population aging, investigating the health impacts of PM_2.5_ and its chemical components in older adults has become increasingly important. Due to age-related limitations in mobility and activity patterns, older adults spend the majority of their time indoors. The chemical composition of PM_2.5_ is complex, and its various components may adversely affect the health of older adults by inducing systemic inflammatory responses. This study examines the association between indoor PM_2.5_ and its components and all-cause mortality, and explores the potential intermediate pathway of systemic inflammatory factors.

**Methods:**

We analyzed 12,607 elderly participants (mean age 86.81 ± 11.34) followed from 2008 to 2018. Over a median follow-up of 4.14 years, 8,643 deaths were recorded. Indoor concentrations of PM_2.5_, black carbon (BC), organic matter (OM), sulfate (SO_4_^2−^), ammonium (NH_4_^+^), and nitrate (NO_3_^−^) were estimated using high-resolution ambient data and infiltration factors. Time-varying Cox proportional hazards models were used to assess the associations between PM_2.5_ and its major components and all-cause mortality. Dose-response relationships were characterized using restricted cubic splines (RCS), and sensitivity analysis with a one-year lag exposure window was also conducted. Additionally, fixed-effects models were used to explore the relationship between PM_2.5_ and the neutrophil-to-lymphocyte ratio.

**Results:**

Time-varying Cox models identified PM_2.5_, BC, OM, and SO_4_^2−^ as significantly associated with increased all-cause mortality. Specifically, a 1 µg/m³ increase in PM_2.5_ was associated with a 0.3% higher risk (*HR* = 1.003, 95% *CI*: 1.001–1.005). Regarding the constituents, each 1 µg/m³ increase in BC corresponded to a 7.2% higher risk (*HR* = 1.072, 95% *CI*: 1.035–1.111); the HRs for OM and SO_4_^2−^ were 1.009 (95% *CI*: 1.002–1.016) and 1.010 (95% *CI*: 1.001–1.019), respectively. These associations remained robust in sensitivity analyses after excluding participants with hypertension or diabetes. Furthermore, the mortality risk was more pronounced among rural residents, and the concentration–risk relationships exhibited a nonlinear pattern. Notably, significant associations were observed between PM_2.5_ and the neutrophil-to-lymphocyte ratio.

**Conclusion:**

This study underscores the need to prioritize control of BC, OM, and SO_4_^2−^ in indoor environments. Stricter emission controls and component-specific standards are needed to reduce health risks.

**Graphical Abstract:**

**Figure:**
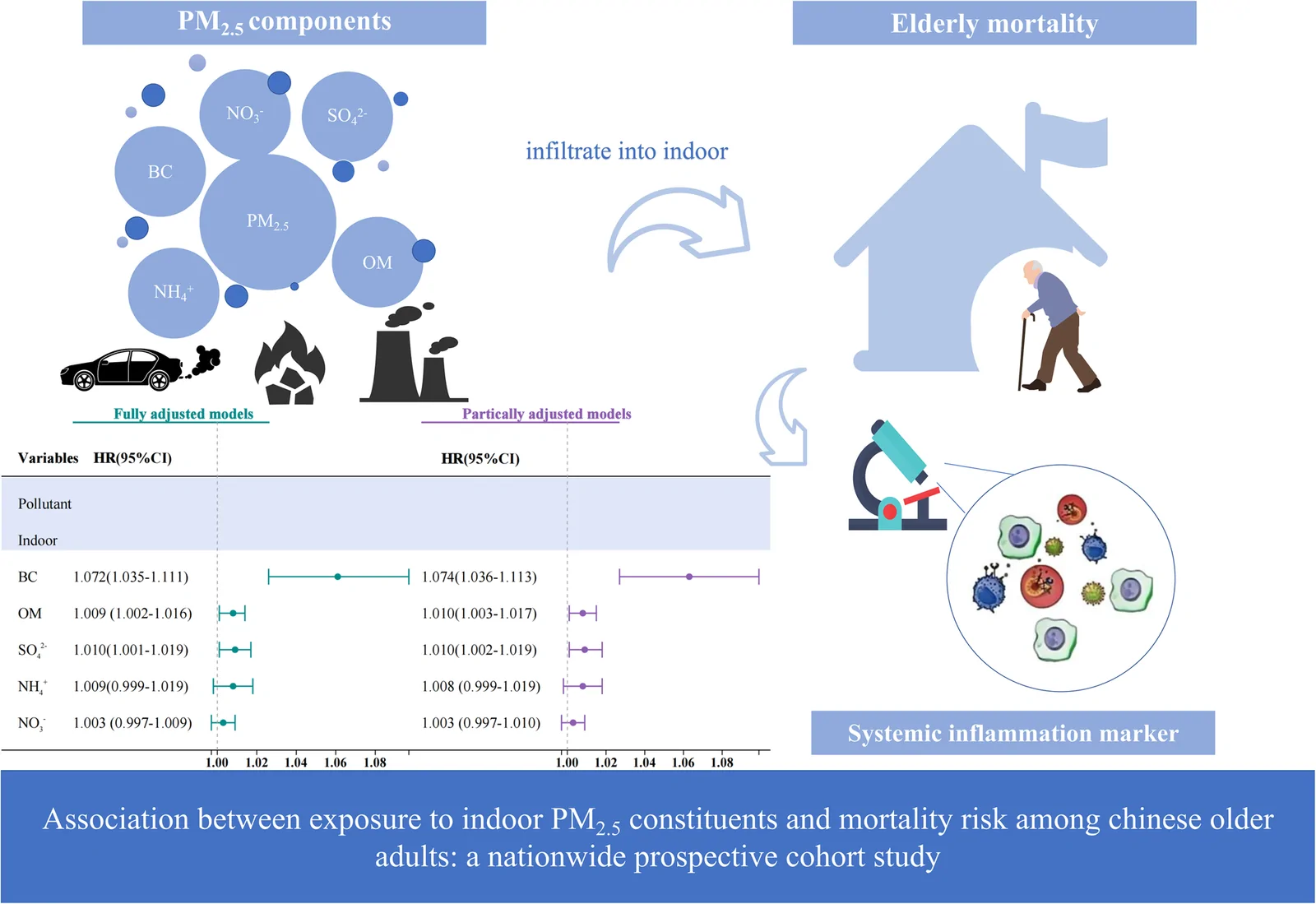

**Supplementary Information:**

The online version contains supplementary material available at https://doi.org/10.1186/s12889-026-29130-1.

## Background

 Air pollution is one of the leading contributors to the global burden of disease and all-cause mortality [1]. In 2023, air pollution is estimated to have caused approximately 7.9 million deaths globally, accounting for roughly one-eighth of total global mortality. Among various air pollutants, ambient fine particulate matter (PM_2.5_, particles with a diameter smaller than 2.5 μm) accounted for approximately 62% of deaths attributable to pollution. Specifically, outdoor PM_2.5_ and indoor air pollution were responsible for 4.9 million and 2.8 million deaths respectively [2]. As a key component of air pollution, particularly in low- and middle-income countries, PM_2.5_ is widely recognized as a major public health threat [3]. A substantial body of epidemiological evidence has demonstrated that PM_2.5_ exposure is strongly associated with increased risks of all-cause and cause-specific mortality [4–6], making it one of the key environmental determinants constraining population health and life expectancy. Older adults are particularly vulnerable to the adverse effects of air pollution due to age-related declines in physiological and immune function, which may contribute to the onset and progression of multiple chronic diseases and, in turn, elevate the risk of premature death [7, 8]. China is currently undergoing the largest and most rapid population aging process in the world. In 2020, the number of adults aged 80 years or older reached 35.8 million, accounting for 2.5% of the total population [9]. The accelerating pace of population aging has heightened the challenge of mitigating the health burden associated with air pollution. The growing environmental susceptibility and disease burden among older adults have emerged as critical public health concerns that require urgent attention [10]. Therefore, elucidating the association between PM_2.5_ exposure and all-cause mortality risk among older populations is of substantial public health importance for promoting sustainable development and healthy aging.

Ambient PM_2.5_ is a complex mixture of multiple components, including black carbon (BC), organic matter (OM; estimated from organic carbon [OC]), sulfate (SO_4_^2-^), nitrate (NO_3_^-^), and ammonium (NH_4_^+^), among others. Most previous studies have focused on PM_2.5_ mass concentration as the exposure indicator and have consistently demonstrated that long-term exposure to higher levels of PM_2.5_ is strongly associated with increased mortality risk [11, 12]. However, by averaging the effects of all components, these studies make it difficult to determine which constituents are most responsible for health risks and may underestimate toxic effects from key components. Notably, a systematic review of 35 studies reported that only BC and OM were significantly associated with natural-cause, cardiovascular, or respiratory mortality and other health outcomes [13]. This indicates that not all components are equally harmful. PM_2.5_ constituents show substantial differences in physicochemical properties, toxic potency, and biological action pathways. These differences may contribute to health impacts in older adults through mechanisms such as systemic inflammation, oxidative stress, and alterations in coagulation function [14–16]. Therefore, focusing on PM_2.5_ components is crucial to identify the key toxic constituents that drive all-cause mortality risks. Such targeted analyses can reduce exposure misclassification, provide evidence for susceptibility in older populations, aid in understanding biological mechanisms, and inform more specific emission control policies. These efforts are important for promoting healthy aging.

PM_2.5_ originates from a diverse array of sources. In regions where solid fuels are not used for cooking and heating, the primary sources are predominantly outdoor, including traffic emissions, power generation, industrial processes, wildfires, and resuspended dust [17]. Although buildings provide some physical shielding, they do not completely prevent the entry of outdoor particles. Outdoor particles can enter indoor environments through natural ventilation via open windows and doors or through cracks and leaks in the building envelope [18]. Field evidence further indicates that window status and building airtightness substantially influence the infiltration of outdoor PM_2.5_ into indoor environments [19]. Current air pollution exposure assessments rely predominantly on outdoor monitoring data but often overlook the fact that, due to functional decline and restricted mobility, older adults spend approximately 85–90% of their time indoors [20]. When exposure in this population is estimated solely from outdoor measurements, the resulting estimates may poorly reflect their true exposure and associated health risks, potentially leading to biased exposure assessment [21–23]. In addition, most existing studies on indoor air pollution have been based on small samples and have used indoor monitoring equipment, and large-scale cohort evidence is still lacking [24]. Although such studies provide valuable individual-level exposure data, they are constrained by limited sample size and representativeness, as well as substantial financial and logistical costs, making it difficult to comprehensively evaluate the long-term health impacts of indoor pollution. A growing body of evidence indicates that indoor air pollution predominantly originates from outdoor sources [25–27]. Therefore, when assessing the health risks to older adults, it is crucial to account for the extent of outdoor pollution penetration into indoor environments. Accordingly, this study aimed to examine the associations between indoor exposure to PM_2.5_ and its major chemical components and all-cause mortality among older adults, and to further investigate whether systemic inflammation may represent a potential pathway underlying these associations.

## Methods

### Participants and procedures

The Chinese Longitudinal Healthy Longevity Survey (CLHLS) is an ongoing, nationwide prospective cohort study designed to investigate determinants of healthy longevity among older adults in China. The survey was initiated in 1998 and subsequently followed up in 2000, 2002, 2005, 2008, 2011, 2014, and 2018. CLHLS uses a multistage, stratified sampling design to provide a nationally representative sample. Participants were recruited from more than 860 counties, county-level cities, or districts across 23 provinces, municipalities, and autonomous regions, covering approximately 85% of the Chinese population. More detailed descriptions of the CLHLS design have been published elsewhere [28–30]. All participants or their legal representatives provided written informed consent before the survey. Trained interviewers conducted face-to-face interviews at participants’ homes using a structured questionnaire to collect information on sociodemographic characteristics, health-related behaviors, and health status. The CLHLS was approved by the Biomedical Ethics Committee of Peking University (IRB00001052-13074).

**Fig. 1:**
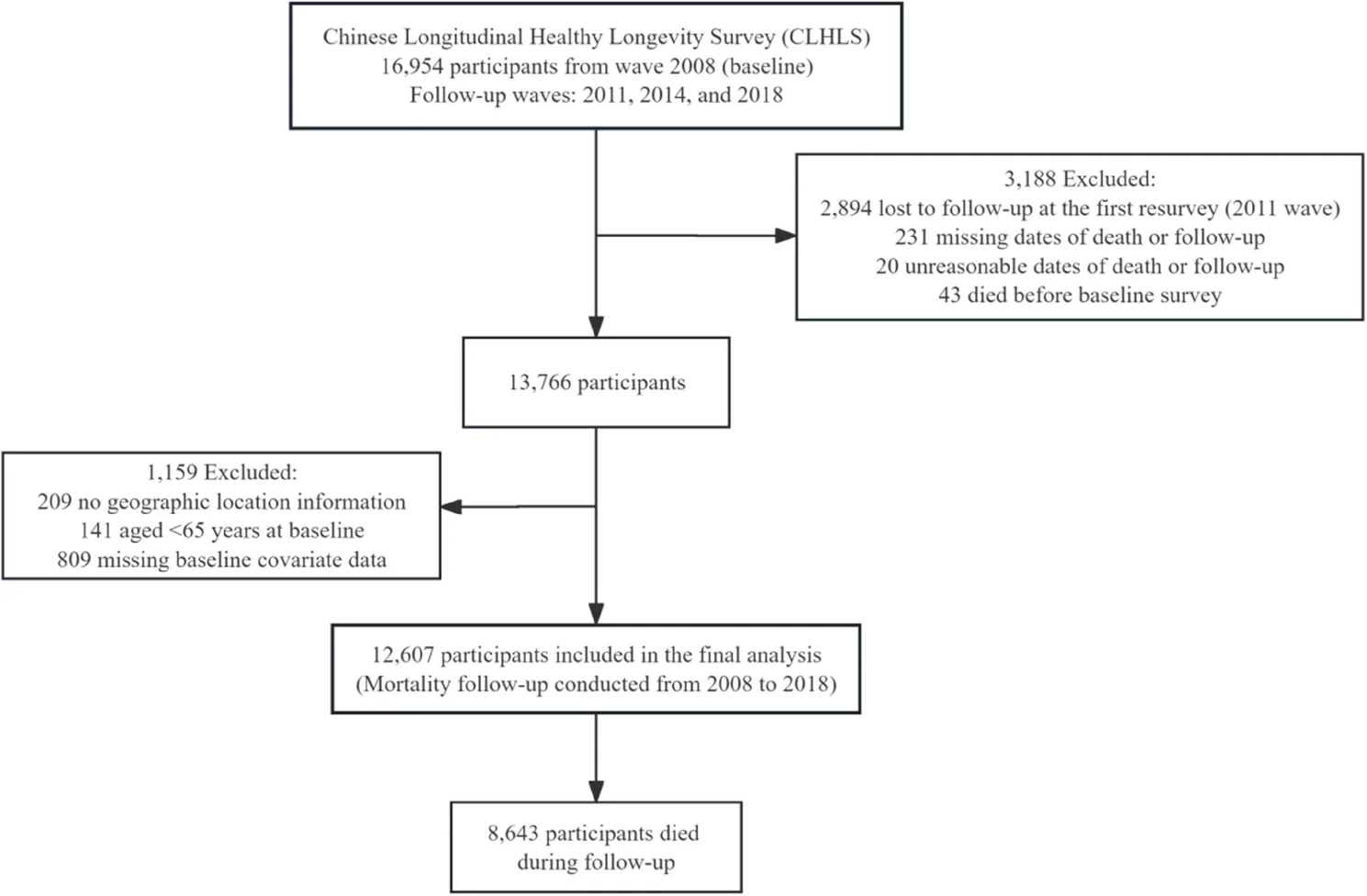
Flowchart of study population selection

**Fig. 2:**
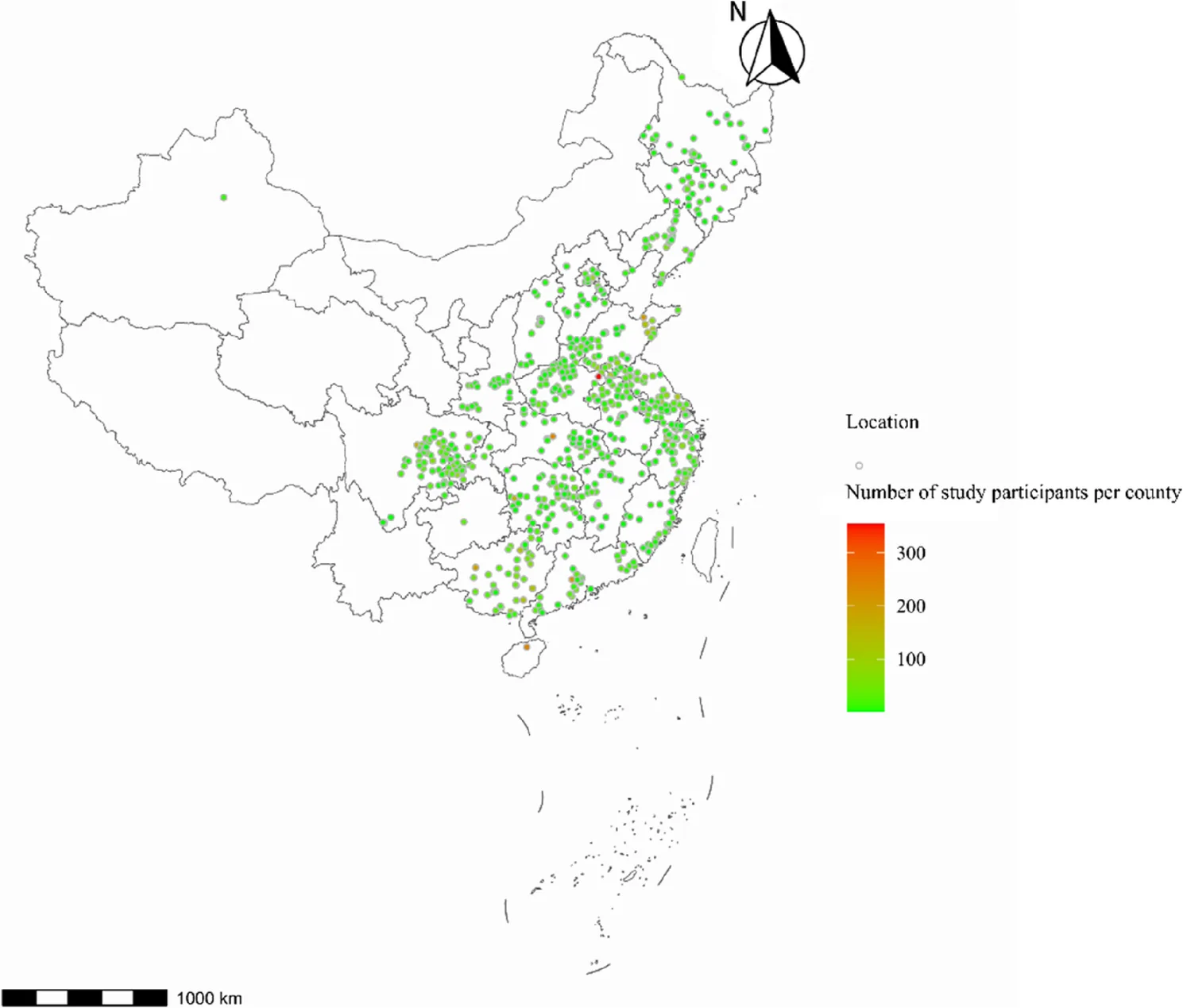
Geographic distribution of study participants across China. (Notes: Each point represents a county included in the study, and the color indicates the number of participants in that county (n), ranging from green (fewer participants) to red (more participants). The relatively sparse distribution in northwestern China reflects its lower population density rather than incomplete geographic coverage.)

In this study, we used CLHLS data from 2008 to 2018. Follow-up began at the baseline survey in 2008 and continued through the subsequent survey waves in 2011, 2014, and 2018, providing a maximum follow-up period of 10 years. We excluded participants who were lost to follow-up at the first resurvey (*n* = 2,894), lacked specific information regarding date of death or had inaccurate death information (*n* = 231 and *n* = 20, respectively), had died before the baseline survey (*n* = 43), had missing residential address information (*n* = 209), were younger than 65 years at baseline (*n* = 141), or lacked baseline covariate data (*n* = 809). Ultimately, 12,607 participants were included in the final analysis (Fig. 1), with the population distribution across cities shown in Fig. 2.

### Environmental exposure

**Fig. 3:**
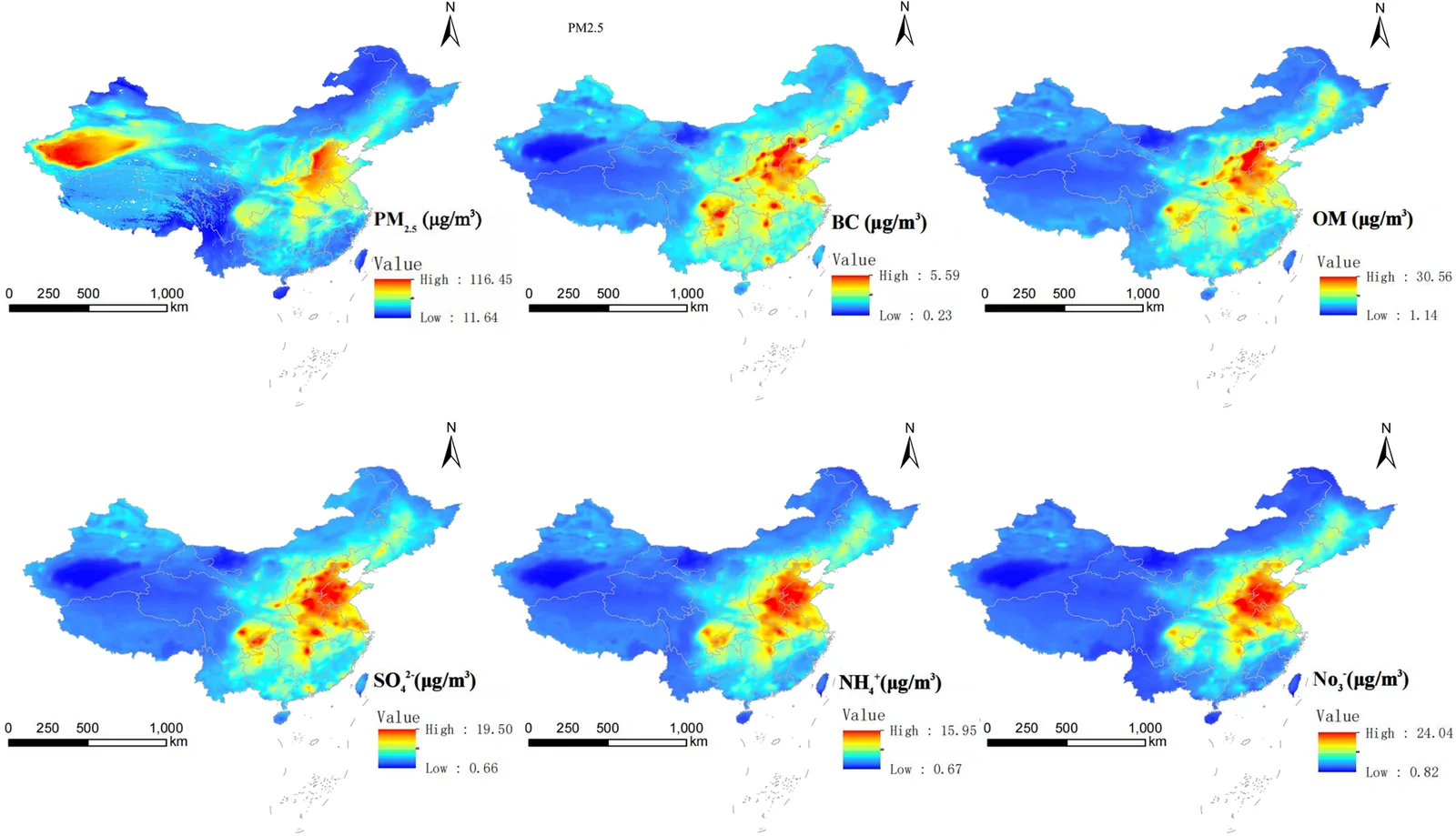
Average exposure concentration distribution of outdoor PM_2.5_ and its major components from 2007 to 2018. Colour scales represent the average concentrations (µg/m^3^), with red indicating higher concentrations and blue indicating lower concentrations

PM_2.5_ data were obtained from the ChinaHighAirPollutants (CHAP) data set, a long-term, full-coverage, 1 km spatial resolution, high-quality dataset of ground-level air pollutants in China. CHAP was generated from big data (e.g., ground-based measurements, satellite remote sensing products, atmospheric reanalysis, and model simulations) using artificial intelligence, considering the spatial and temporal heterogeneity of air pollution [31]. Annual raster data for PM_2.5_ chemical components (BC, OM, SO_4_^2-^, NH_4_^+^, NO_3_^-^) were obtained from the Tracking Air Pollution (TAP) database in China (temporal resolution: annual; spatial resolution: 10 × 10 km; source: http://tapdata.org.cn/) [32, 33]. In the TAP dataset, OM was derived from measured OC concentrations rather than directly measured. When a source-specific OM/OC ratio was unavailable, an OM/OC conversion factor of 1.6 was applied to estimate OM [32]. TAP integrates multiple sources, including satellite remote sensing, machine learning algorithms, ground monitoring, atmospheric reanalysis, and model simulations, to generate high-resolution near-surface air pollution data. The predicted PM_2.5_ and its major components concentrations have shown good consistency with ground-based measurements, with validation metrics as follows: PM_2.5_ cross-validation coefficient of determination (CV-R^2^) = 0.92, root mean square error (RMSE) = 10.76 µg/m^3^, mean absolute error (MAE) = 6.32 µg/m^3^. The correlation coefficients of 0.75, 0.70, 0.75, 0.64, and 0.72 for NO_3_^-^, SO_4_^2-^, NH_4_^+^, BC, and OM, respectively [32, 33]. Figure 3 shows the regional distribution of outdoor PM_2.5_ and its major components.

To estimate the concentration of indoor PM_2.5_ and its major components, we adopted the 0.61 infiltration factor reported by Krebs et al., based on large-scale observational data [34]. This factor is derived from over 1,000 crowdsourced data points in California and is the first large-scale study to explore the temporal and spatial relationships between outdoor and indoor particulate matter pollution across particle sizes. Compared with previous studies that only focused on a few research sites and had relatively short observation periods, this study’s value represents a relatively high estimate of the conversion of outdoor particulate matter concentration to indoor concentration, and the penetration effect on fine particulate matter is more significant, in line with the conservative principle commonly adopted in health risk assessment. Krebs et al. combined basic factors such as meteorology, monitoring point and time fixed effects, and also included heterogeneous factors such as building, climate, temperature, and particle size to obtain this outdoor-indoor penetration factor, which helps us better separate the biological effects from the concentration-response function. By applying this parameter, we aim to provide a relatively robust basis for indoor exposure assessment, ensuring the scientific validity and rationality of exposure assessment in environmental health impact assessment. This conservative approach helps avoid underestimating exposure levels and provides more compelling evidence for policymakers to promote the implementation of air pollution control measures.

To evaluate long-term exposure for each participant, we used refined spatial and temporal resolutions. Spatially, we first aggregated the environmental data to the county level using the administrative boundaries of 781 cities, then calculated the average concentration for each county unit. It is important to note that due to privacy protection regulations, personal residential address information cannot be obtained. In this case, environmental exposure values at the district/county level were assigned to each participant based on the six-digit postal code of their residential location. Therefore, participants living in the same county/district were assigned to the same exposure level. The unique six-digit postal codes were identified using the 2010 Chinese county census data [35]. For each survey wave, exposure was defined as the average pollutant concentration during the survey year and the preceding year. This two-year averaging window was selected to minimize year-to-year variability potentially driven by unusual meteorological conditions, thereby providing a more stable measure of recent cumulative exposure than a single-year average. Similar exposure averaging approaches have been applied in previous cohort studies investigating PM_2.5_ exposure and mortality [12, 36].

### Outcome

The primary outcome of this study was all-cause mortality. During the follow-up surveys, the survival status of the participants was classified as alive, deceased, or lost to follow-up. Mortality information, including the exact calendar date of death, was ascertained from family members (children or spouses) or primary caregivers, with death certificates used for verification when available. When mortality information could not be obtained from these sources, further verification was conducted through local community committees. Follow-up time was calculated as the interval between the first interview and either the last interview or date of death.

### Covariates

The analysis adjusted for covariates across four domains. (1) Demographic characteristics: age, sex(male/female), residence (rural/urban), marital status (partnered/other), educational attainment (illiterate/literate, literate defined as literate with ≥1year of schooling, else illiterate). (2) Health behaviours: current smoking status (yes/no), current alcohol consumption (yes/no), engagement in regular exercise (yes/no), self-reported sleep quality (very good, good, fair, poor, very poor), and pension receipt (yes/no). (3) Health conditions: hypertension (yes/no), diabetes (yes/no), and body mass index (BMI), categorized as underweight (< 18.5 kg/m^2^), normal weight (18.5–23.9 kg/m^2^), overweight (24–27.9 kg/m^2^), obese (≥ 28 kg/m^2^) [37–42]. (4) Temperature exposure: ambient temperature data were obtained from the TAP database at a 10 × 10 km spatial and daily temporal resolution. To estimate indoor exposure, outdoor grid temperatures were converted using the validated piecewise linear regression by Hou et al. [43], based on the American Society of Heating, Refrigerating, and Air-Conditioning Engineers (ASHRAE) Global Thermal Comfort Database to convert outdoor temperature grid data into corresponding indoor temperature estimates. As ambient temperature is a significant confounder that directly influences pollutant concentrations and independently affects mortality [44], it was controlled for in all models.

### Statistical methods

Baseline characteristics were summarized using descriptive statistics. Categorical variables were reported as frequency (*n*) and percentage (*%*). Continuous variables were assessed for normality using the Kolmogorov-Smirnov test; those normally distributed were described as mean ± standard deviation ($\:\stackrel{-}{X}$±SD). Time-varying Cox regression models were employed to assess the association between indoor PM_2.5_ and its major components and all-cause mortality risk, using survey time points as the temporal scale and incorporating time-varying covariates [45–47]. Two models were fitted: a fully adjusted model controlling all prespecified confounders, and a partially adjusted model that excluded hypertension and diabetes. In addition to the analyses based on estimated indoor PM_2.5_ and its components, outdoor PM_2.5_ and its components were also directly evaluated as independent exposure variables in separate models. This exclusion was based on scientific considerations: hypertension and diabetes are not only common chronic diseases among the elderly but may also represent key intermediate pathological pathways through which PM_2.5_ induces inflammation and ultimately leads to mortality. Comparing the effect estimates of both models helps assess the robustness of the results [48–50]. To mitigate the impact of collinearity on the estimated association between multiple-pollutant co-exposure and all-cause mortality, a regression-based approach was applied to highly correlated PM_2.5_ components, with the resulting residuals incorporated as covariates in the model [51].

This study further explored potential effect modification of the associations between indoor PM_2.5_ and its major components and all-cause mortality risk. Stratified analyses were conducted according to residential location (rural or urban), sex (male or female), and age groups (≤ 90 years, 91–100 years, and > 100 years) to evaluate whether the magnitude of associations differed across demographic characteristics. Effect modification was formally assessed by incorporating multiplicative interaction terms between each pollutant and subgroup variable into fully adjusted Cox proportional hazards models. The age grouping was informed by prior research and considered that the very elderly, due to declining physical function and significantly increased time spent indoors, may be more susceptible to indoor PM_2.5_ exposure [10, 29].

Furthermore, this study employed restricted cubic splines (RCS) to fit the exposure-response relationship between indoor PM_2.5_ and its major components and all-cause mortality risk, with statistical testing of nonlinear terms conducted via likelihood ratio tests (*α* = 0.05). For sensitivity analyses, we refitted the model using one-year lagged exposure concentrations to assess the robustness of the primary findings to alternative exposure windows [39]. We also conducted a sensitivity analysis using a lower infiltration factor of 0.41, derived from the lower bound of the annual averaged PM_2.5_ infiltration factor estimated by Shi et al. in naturally ventilated residences in Beijing (0.48 ± 0.07), to evaluate the robustness of the observed associations under an alternative indoor exposure estimation scenario [52].

To complement the mortality analysis and further examine the relationship between PM_2.5_ exposure and systemic inflammation, we used two waves of biomedical data from the CLHLS collected in 2008 and 2014. The analysis included 273 participants with repeated biomedical measurements, contributing 546 person-wave observations. For each survey wave, PM_2.5_ exposures were calculated as the average concentrations during the survey year and the preceding year, consistent with the exposure assessment used in the main analysis. We applied fixed-effects models to examine the associations of indoor and outdoor PM_2.5_ concentrations with log-transformed derived neutrophil-to-lymphocyte ratio (dNLR). The models were adjusted for BMI, marital status, residence, smoking, alcohol consumption, regular exercise, hypertension, and diabetes, as well as the corresponding indoor or outdoor temperature. The dNLR was calculated as (white blood cell count-lymphocyte count)/lymphocyte count and was used as a marker of systemic inflammation levels [53].

All statistical analyses were conducted using R (version 4.5.1), and a 2-tailed *P* < 0.05was considered statistically significant.

## Results

This study included 12,607 elderly participants, whose baseline characteristics are detailed in Table 1. The mean age of the participants was 86.81 ± 11.34 years, with females accounting for 56.68%. Throughout the decade of follow-up, 8,643 death events were documented, corresponding to an overall all-cause mortality rate of 68.30%. Univariate analysis showed that, compared with the survival group (*n* = 3,964), the death group (*n* = 8,643) had a significantly higher proportion of females, individuals without a spouse, rural residents, individuals without a pension, and individuals with a lower body mass index (all *p* < 0.001).

**Table 1:** Baseline characteristics of study participants

Varibles	Total (*n* = 12,607)	Survivors (*n* = 3,964)	Deaths (*n* = 8,643)	*p*-value
Demographic characteristics				
Age	86.81 ± 11.34	77.83 ± 9.64	90.92 ± 9.55	< 0.001
Sex				
Male	5,461(43.32)	1,826(46.06)	3,635(42.06)	< 0.001
Female	7,146(56.68)	2,138(53.94)	5,008(57.94)	
Education				
Literate	4,679(37.11)	1,948(49.14)	5,980(69.16)	< 0.001
Illiterate	7,928(62.89)	2,016(50.86)	2,663(30.81)	
Marital status				
Married	3,984(31.60)	2,116(53.38)	1,868(21.61)	< 0.001
Not married	8,623(68.40)	1,848(46.62)	6,775(78.39)	
Residence				
Urban	4,699(37.27)	1,674(42.23)	3,025(35.00)	< 0.001
Rural	7,908(62.73)	2,290(57.77)	5,618(65.00)	
Behavioral factors				
Smoking				
Yes	2,294(18.20)	855(21.57)	1,439(16.65)	< 0.001
No	10,313(81.80)	3,109(78.43)	7,204(83.35)	
Drinking				
Yes	2,271(18.01)	815(20.56)	1,456(16.85)	< 0.001
No	10,336(81.99)	3,149(79.44)	7,187(83.15)	
Regular exercise				
Yes	3,494(27.71)	1,529(38.57)	1,965(22.74)	< 0.001
No	9,113(72.29)	2,435(61.43)	6,678(77.26)	
Quality of sleep				
Very good	95(0.75)	35(0.88)	60(0.69)	< 0.001
Good	1,136(9.01)	397(10.02)	739(8.55)	
Fair	2,973(23.58)	944(23.81)	2,029(23.48)	
Poor	6,734(53.41)	1,981(49.97)	4,753(54.99)	
Very poor	1,669(13.24)	607(15.31)	1,062(12.29)	
Retirement pension				
Yes	1,928(15.29)	3,046(76.84)	7,633(88.31)	< 0.001
No	10,679(84.71)	918(23.16)	1,010(11.69)	
Health status				
BMI				
Obesity	341(2.70)	164(4.14)	177(2.05)	< 0.001
Overweight	1,392(11.04)	650(16.40)	742(8.58)	
Underweight	4,143(32.86)	905(22.83)	3,238(37.46)	
Normal	6,731(53.39)	2,245(56.63)	4,486(51.90)	
Hypertension				
Yes	2,460(19.51)	919(23.18)	1,541(17.83)	< 0.001
No	10,147(80.49)	3,045(76.82)	7,102(83.17)	
Diabetes				
Yes	294(2.33)	126(3.18)	168(1.94)	< 0.001
No	12,313(97.67)	3,838(96.82)	8,475(98.06)	
Indoor environmental status				
Temperature	22.98 ± 2.83	23.03 ± 2.89	22.96 ± 2.81	0.227
PM_2.5_	37.55 ± 9.57	37.45 ± 9.67	37.60 ± 9.52	0.424
BC	2.21 ± 0.65	2.22 ± 0.65	2.20 ± 0.65	0.170
OM	9.99 ± 3.27	10.04 ± 3.22	9.96 ± 3.29	0.207
SO_4_^2−^	7.91 ± 2.56	7.94 ± 2.51	7.90 ± 2.58	0.369
NH_4_^+^	5.73 ± 2.07	5.70 ± 1.99	5.75 ± 2.11	0.229
NO_3_^−^	7.79 ± 3.40	7.79 ± 3.25	7.79 ± 3.47	0.996

**Fig. 4:**
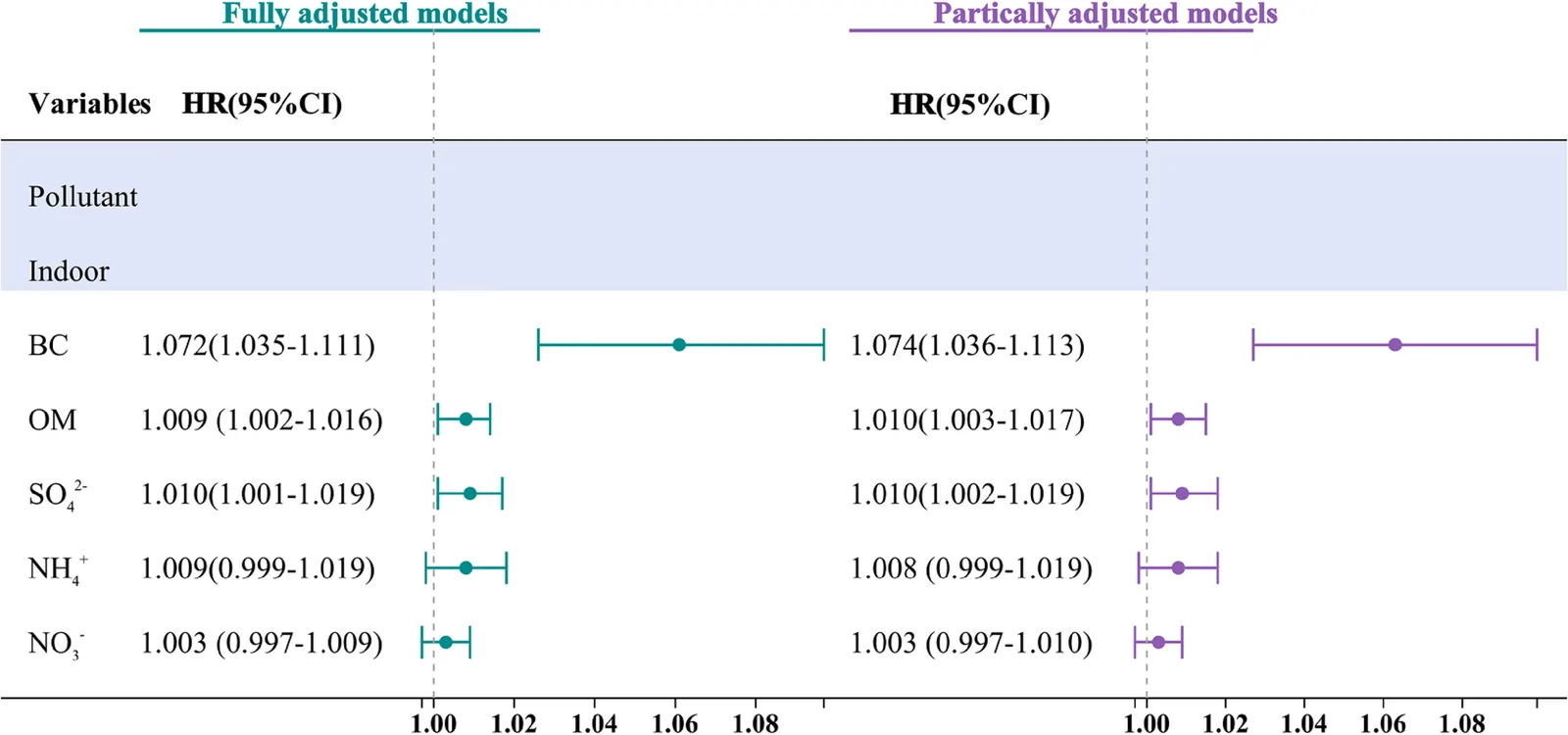
Random forest plots of fully and partially adjusted models

This study systematically evaluated the associations between indoor PM_2.5_ and its major components and all-cause mortality risk using both fully adjusted and partially adjusted models. Results shown in Fig. 4 indicated a significant positive association between indoor PM_2.5_ and all-cause mortality risk. In the fully adjusted model, each 1 µg/m³ increase in indoor PM_2.5_ concentration was associated with a 0.3% higher mortality risk (*HR* = 1.003, 95% *CI*: 1.001–1.005). Similarly, in the partially adjusted model, after excluding hypertension and diabetes, the association remained stable (*HR* = 1.003, 95% *CI*: 1.001–1.005), indicating high reliability of the results. Moreover, BC, OM, and SO_4_^2−^ were all significantly positively associated with mortality risk in the fully adjusted model, with hazard ratios of 1.072 (95% *CI*: 1.035–1.111), 1.009 (95% *CI*: 1.002–1.016), and 1.010(95% *CI*: 1.001–1.019) per 1 µg/m^3^ increase in their concentrations, respectively. In contrast, neither NH_4_^+^ nor NO_3_^−^ was significantly associated with mortality risk. In comparison, the associations for outdoor pollutants differed markedly. In the fully adjusted model, only outdoor BC was significantly associated with all-cause mortality risk (*HR* = 1.040, 95% *CI*: 1.017–1.063). In the partially adjusted model, outdoor BC remained significant (*HR* = 1.041, 95% *CI*: 1.018–1.064). No significant associations were observed for outdoor OM, SO_4_^2−^, NH_4_^+^, NO_3_^−^, or PM_2.5_ in either model. These results are detailed in Supplementary Material Table 2. Sensitivity analyses using an alternative infiltration factor of 0.41 showed that the main associations remained robust compared with those obtained using an infiltration factor of 0.61. Specifically, the positive associations of indoor PM_2.5_, BC, OM, and SO_4_^2−^ with mortality risk remained statistically significant, whereas NH_4_^+^ and NO_3_^−^ remained non-significant (Supplementary Material Table 9).

**Fig. 5:**
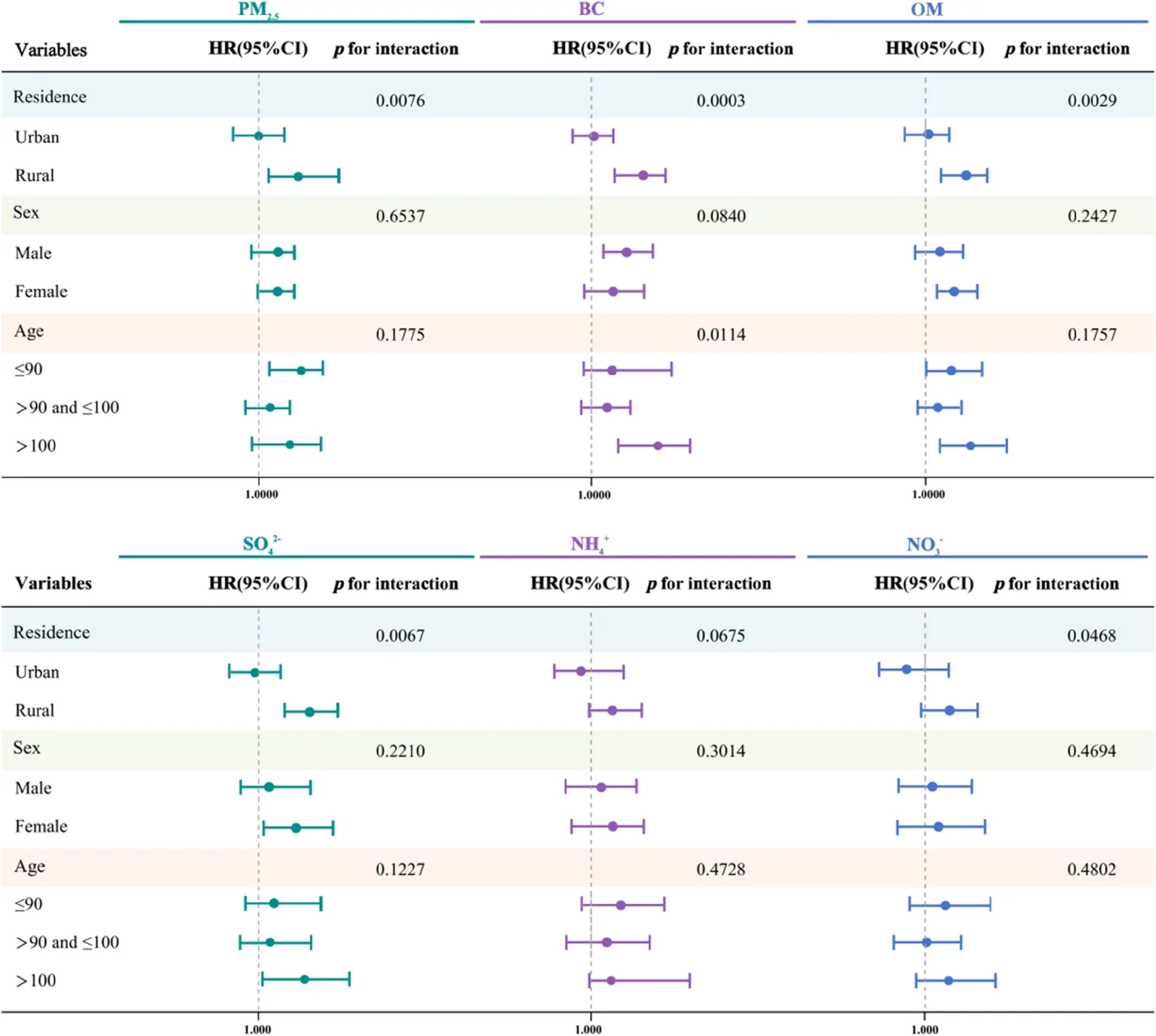
Subgroup analyses and interaction tests for the associations between indoor PM_2.5_ components and all-cause mortality risk

To further evaluate whether the associations between indoor PM_2.5_ and its major components and all-cause mortality risk differed across population subgroups, analyses were stratified by residential location, sex, and age group, with formal interaction tests performed to assess potential effect modification (Fig. 5). Significant effect modification by residential location was observed for indoor PM_2.5_, BC, OM, and SO_4_^2−^ (all *p* for interaction < 0.01). In subgroup analyses, stronger associations were observed among rural residents. Specifically, indoor PM_2.5_ was significantly associated with mortality risk among rural participants (*HR* = 1.005, 95% *CI*: 1.002–1.008). A similar association was observed for BC (*HR* = 1.124, 95% *CI*: 1.069–1.182), OM (*HR* = 1.016, 95% *CI*: 1.006–1.025), and SO_4_^2−^ (*HR* = 1.017, 95% *CI*: 1.005–1.028). No statistically significant differences were observed between sex subgroups with respect to indoor PM_2.5_ or its major components (all *p* for interaction > 0.05). For age groups, the strongest association between BC exposure and mortality risk was observed among participants aged > 100 years (*HR* = 1.158, 95% *CI*: 1.075–1.247; *p* for interaction = 0.0114). However, no statistically significant age-related effect modification was detected for indoor PM_2.5_, OM, NH_4_^+^, NO_3_^−^, or SO_4_^2−^ (all *p* for interaction > 0.05).

Among 273 participants with repeated biomedical measurements (546 person-wave observations), individual fixed-effects models showed statistically significant positive associations of both indoor and outdoor PM_2.5_ concentrations with log-transformed dNLR (Supplementary Material Table 1). These findings indicate that within-individual increases in PM_2.5_ exposure were associated with higher dNLR levels.

We examined the association between co-exposure to PM_2.5_ components and all-cause mortality risk in an elderly population. Notably, there was a highly significant positive correlation between BC, OM, SO_4_^2−^, NH_4_^+^, and NO_3_^−^ (Supplementary Materials Fig. 1), indicating that they have similar sources and coexist in the air. Building on this observation, after adjusting for potential residual confounding, we evaluated the association between combined exposure to multiple indoor PM_2.5_ components and all-cause mortality (Supplementary Materials Table 3). The finding was consistent with single-pollutant models, showing robust statistical associations of BC, OM, and SO_4_^2−^ with mortality. Furthermore, this study assessed the associations between combined exposures to outdoor PM_2.5_ components and all-cause mortality, with results showing a significant association for BC (Supplementary Materials Table 4). Further, in one-year lagged models, BC remained significantly associated with all-cause mortality risk across all exposure sources (indoor/outdoor) and types (single/combined) (Supplementary Materials Tables 5-Table 8).

**Fig. 6:**
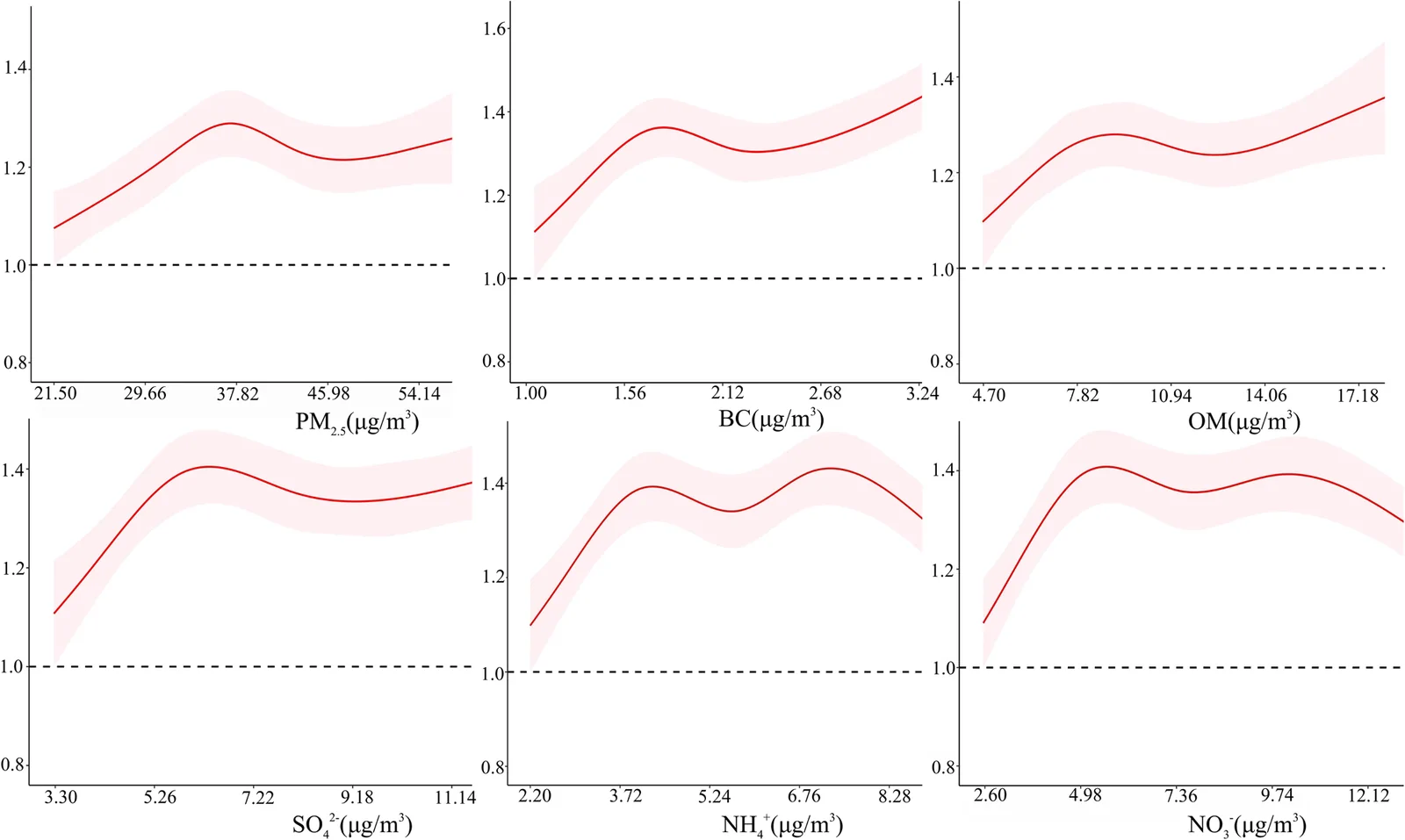
Analysis of the nonlinear association between indoor pollutants and mortality risk

To explore potential nonlinear associations, we employed RCS to model the dose-response relationships between indoor PM_2.5_ and its major components and all-cause mortality risk, with full adjustment for confounders (demographic characteristics, behavioral factors, health status, and indoor temperature). Likelihood ratio tests showed significant nonlinear trends for all exposures examined (All Nonlinear *p* < 0.05; Fig. 6). The shapes of the exposure-response curves differed across pollutants. For indoor PM_2.5_, BC, OM, and SO_4_^2−^, the curves displayed an increasing pattern with an initial rise, a plateau, and a further increase at higher concentrations. For NH_4_^+^ and NO_3_^−^, the curves exhibited non-monotonic associations, featuring wave-like fluctuations with multiple turning points across the concentration range.

## Discussion

This study systematically evaluated the association between PM_2.5_ and its major components, including BC, OM, NO_3_^−^, NH_4_^+^, and SO_4_^2−^, and all-cause mortality risk among the elderly, based on a representative large-scale prospective cohort study from China. The results showed that PM_2.5_, BC, OM, and SO_4_^2−^ were associated with an increased risk of death. Subgroup analyses revealed greater susceptibility among rural residents. Additionally, fixed-effects models demonstrated significant positive associations of PM_2.5_ with dNLR. Moreover, restricted cubic spline analyses revealed significant nonlinear exposure-response associations between indoor PM_2.5_ and its major components and mortality risk, suggesting that the magnitude of the associations varied across exposure ranges. The robustness of these findings was confirmed by sensitivity analyses.

In this study, we focused on the impact of outdoor pollutants penetrating indoors on all-cause mortality risk in the elderly. This consideration is based on the fact that the elderly population, due to physical degeneration and limited mobility, typically spends the majority of their time indoors. Our findings revealed the contribution of indoor PM_2.5_ and its major components to all-cause mortality risk, particularly in a large-scale population such as China’s. As our analysis transitioned from outdoor to indoor environments, a distinct finding emerged: the spectrum of statistically significant pollutants expanded beyond BC to also include OM and SO_4_^2−^. This expanded range of significant indoor components aligns with previous studies on the health effects of outdoor PM_2.5_ constituents and addresses a critical gap in understanding the health implications of indoor exposure. To provide further context, prior evidence has shown that long-term exposure to ambient PM_2.5_ components is associated with elevated mortality. For instance, a prospective cohort study based on 30,524 adults from the China Health and Nutrition Survey reported associations of ambient PM_2.5_ and its major components with all-cause mortality, with nonlinear relationships observed for OM and NH_4_^+^ [54]. Based on an analysis of U.S. Medicare data, a study of beneficiaries aged 65 and older found that elevated concentrations of six ambient PM_2.5_ components, including BC, OM, NO_3_^−^, NH_4_^+^, SO_4_^2−^, and dust, were associated with increased all-cause mortality, with effect estimates increasing progressively with age [14]. Collectively, these studies provide a foundational basis for our work and mutually underscore the consistency and reproducibility of our findings. Ultimately, this highlights the importance of incorporating indoor exposure assessment in future air pollution and health research.

The differential associations observed for specific PM_2.5_ components in this study suggest that component-specific toxicological properties may contribute to the observed all-cause mortality patterns. BC showed the strongest association with mortality, whereas OM and SO_4_^2−^ also demonstrated significant associations in indoor exposure models, highlighting the importance of considering PM_2.5_ composition rather than mass concentration alone. BC, a carbonaceous product of incomplete combustion, has a large specific surface area and strong adsorption capacity, allowing it to carry redox-active compounds and transition metals that may enhance oxidative stress responses [55, 56]. After respiratory deposition, BC may activate pulmonary immune cells and induce persistent oxidative stress and inflammatory responses, which may partly explain its stronger association with mortality observed in this study [57]. OM contains biologically active compounds, including polycyclic aromatic hydrocarbons and quinone derivatives, which may contribute to oxidative stress, mitochondrial dysfunction, and cellular injury [58, 59]. Together, these mechanisms provide biological plausibility for the observed associations between specific indoor PM_2.5_ components and mortality risk and support the potential role of oxidative stress and inflammatory pathways in driving these effects.

To further examine the relationship between PM_2.5_ exposure and systemic inflammation, we applied individual fixed-effects models to analyze the associations of indoor and outdoor PM_2.5_ concentrations with dNLR. Both indoor and outdoor PM_2.5_ concentrations were significantly and positively associated with dNLR, providing preliminary epidemiological evidence of a relationship between PM_2.5_ exposure and systemic inflammation. A population-based cohort study reported that a higher neutrophil-to-lymphocyte ratio was associated with increased risks of all-cause and cardiovascular mortality in a predominantly older general population [60]. Sustained low-grade inflammation has also been associated with impaired cardiovascular, respiratory, and immune function and with increased risks of related diseases and mortality [61–63]. In summary, these findings suggest that systemic inflammation could be a potential biological process relevant to the adverse health effects of PM_2.5_ exposure. However, our findings represent only a preliminary exploration of the potential inflammatory mechanisms linking PM_2.5_ exposure and all-cause mortality. Further experimental studies and large prospective cohort studies with more comprehensive inflammatory biomarkers are warranted to validate these findings and elucidate the underlying biological mechanisms.

Significant residence-related effect modification was observed, with generally stronger associations between indoor PM_2.5_ components and all-cause mortality among rural residents. In rural areas of China, elevated indoor BC exposure may partly reflect the continued use of solid fuels and biomass combustion for household energy, including wood, crop residues, coal, and other polluting fuels. Organic matter and sulfate mainly originate from fossil fuel combustion [64, 65]. This disparity may be further influenced by differences in socioeconomic conditions, environmental awareness, and access to healthcare resources, which could modify population susceptibility to environmental exposures [66, 67]. An assessment using a mobile, taxi-based monitoring system found that PM_2.5_ concentrations in some rural areas of China, exceed urban levels, suggesting chronic exposure of rural people to highly polluted environments [68]. Similar urban-rural disparities exist in other developing countries, such as the higher proportion of premature deaths attributable to PM_2.5_ in rural areas of India’s Saharanpur region [69]. Even in developed countries like the United States [70] and South Korea [71], spatial heterogeneity in air pollution’s health impacts persists, indicating that regionally concentrated high-exposure pollution remains a global challenge.

This study identified significant age-related effect modification for indoor BC, with the association observed among participants aged > 100 years. This heterogeneity may reflect the combined influence of cumulative environmental exposure, age-related declines in physiological function, and reduced resilience to environmental stressors among extremely old adults [72]. Given the high underlying all-cause mortality risk and complex health conditions in this age group, these findings should be interpreted cautiously. Age-related declines in pulmonary function, immune regulation, and the clearance capacity for inhaled particles may further reduce the ability of extremely old adults to tolerate environmental stressors such as BC exposure [73, 74]. Importantly, a previous study identified BC as an important contributor to PM_2.5_-related mortality risk among Chinese oldest-old adults, which is consistent with our findings and supports the potential role of BC in mortality risk among extremely old adults exposed to indoor air pollution [10]. However, given the observational nature of the present study and the complexity of mortality determinants in this age group, further studies in independent cohorts are warranted to validate these age-specific associations and clarify the contribution of BC exposure to all-cause mortality risk among extremely old populations.

The nonlinear exposure–response patterns observed in this study provide additional insights into the complexity of the associations between PM_2.5_ components and all-cause mortality risk. Indoor PM_2.5_, BC, OM, and SO_4_^2−^ each showed increasing associations with all-cause mortality risk across most of the exposure range, with the curves exhibiting nonlinear shapes. These nonlinear exposure-response curves enable the identification of concentration ranges with pronounced increases in mortality risk and potential plateau regions, offering evidence for risk assessment and intervention strategies [14, 75]. For NH_4_^+^ and NO_3_^−^, the exposure–response curves exhibited non-monotonic patterns with fluctuations across the concentration range, consistent with the heterogeneous concentration–response relationships documented for different PM_2.5_ constituents [76]. These non-monotonic patterns may partly reflect compositional effects, as changes in the relative proportion of NH_4_^+^ and NO_3_^−^ within the PM_2.5_ mixture have been shown to modify the predicted mortality risk [77]. The semivolatile nature of ammonium nitrate may also add complexity to exposure assessment and influence the curve shape at higher exposure concentrations [78]. Further toxicological and mechanistic studies are needed to clarify the biological pathways underlying these nonlinear exposure-response relationships.

The accelerating pace of global population aging elevates indoor air quality to a critical modifiable determinant of elderly health [79]. Marked disparities in both exposure levels and biological susceptibility persist worldwide, with the most pronounced inequities observed in rural areas of developing countries and heavily polluted regions within developed nations. These disparities necessitate that policymakers implement systematic environmental and health interventions targeting high-risk zones to establish equitable health safeguards. In aging societies, intervention strategies must be tailored to accommodate the distinct exposure patterns of susceptible subgroups, particularly rural residents and older adults confronting indoor pollution risks [80, 81]. Concrete measures include integrating indoor air quality standards into long-term care frameworks and elderly-oriented building codes, mandating high-efficiency ventilation systems in renovation projects, promoting the adoption of indoor air purifiers through subsidy programs, and incorporating indoor environmental exposure assessments into routine elderly health management protocols. Collectively, such interventions hold promise for effectively reducing exposure, slowing environmentally mediated functional decline, and advancing the overarching goal of “healthy aging” [82].

This study has several key strengths: (1) The use of a nationally representative, large-scale prospective cohort study from the China Health and Longevity Survey (23 provinces, > 10,000 elderly participants, up to 10-year follow-up), which supports result generalizability and the evaluation of long-term cumulative exposure; (2) A focus on specific indoor PM_2.5_ chemical components (BC, OM, SO_4_^2−^, NO_3_^−^, NH_4_^+^), overcoming the common limitation of relying solely on outdoor or total mass concentration data, thereby providing direct evidence on critical indoor toxicants; (3) A multilevel evidence chain linking population health risks to inflammatory mechanisms was established. The fixed-effect model confirmed the association of PM_2.5_ and dNLR, with findings consistent with established toxicological mechanisms; (4) The identification through subgroup analyses of susceptible subpopulations, which informs targeted public health strategies. However, this study is subject to the following limitations. (1) Indoor pollutant concentrations were derived from model estimates without considering the effects of indoor wind speed and indoor pollutant emissions, which may lead to bias in exposure estimation. (2) Despite adjustment for a range of covariates, the possibility of residual confounding persists, especially due to unmeasured lifestyle and socioeconomic variables.

## Conclusion

This study demonstrates that PM_2.5_ infiltration into indoor environments remains significantly associated with elevated all-cause mortality risk among older adults, particularly among those exposed to BC, OM, and SO_4_^2−^. This population-level finding is supported by elevated, consistent with established toxicological mechanisms. These results underscore the necessity for stricter emission controls and targeted interventions, especially for vulnerable subgroups such as rural residents. In the future, prioritizing the mitigation of BC, OM, and SO_4_^2−^ should be central to air quality management policies. Both China and the international community need to establish refined, component-specific standards for particulate matter to help reduce the all-cause mortality burden associated with PM_2.5_ exposure and alleviate the public health challenges posed by population aging.

## Supplementary Information

Supplementary Material 1.

## Data Availability

The datasets analysed during the current study are available in the Peking University Open Access Research Database, [https://opendata.pku.edu.cn/dataverse/CHADS].

## Abbreviations

PM_2.5_: Particulate matter with a diameter smaller than 2.5 μm
BC: Black carbon
OM: Organic matter
SO_4_^2−^: Sulfate
NO_3_^−^: Nitrate
NH_4_^+^: Ammonium
CLHLS: The Chinese Longitudinal Healthy Longevity Survey
CI: Confidence interval
HR: Hazard ratio
CHAP: ChinaHighAirPollutants
TAP: Tracking Air Pollution
CMAQ: The community multiscale air quality model
ASHRAE: American Society of Heating, Refrigerating, and Air-Conditioning Engineers
RCS: Restricted cubic splines
dNLR: Derived neutrophil-to-lymphocyte ratio
NADPH: Nicotinamide adenine dinucleotide phosphate
ROS: Reactive oxygen species
COVID-19: Coronavirus Disease 2019
