# Supplementary material for: Association between exposure to indoor PM2.5 constituents and mortality risk among chinese older adults: a nationwide prospective cohort study
Source: BMC Public Health. 2026 Sep 7;26:2859. doi: 10.1186/s12889-026-29130-1 (PMC13644256; doi:10.1186/s12889-026-29130-1)
Supplement: Supplementary file 1 [file 12889_2026_29130_MOESM1_ESM.docx]

**Supplementary Materials：**

**contents:**

**Supplemental [Table1	2](#_Toc22729)**

**Supplemental [Table2	3](#_Toc11123)**

**Supplemental [Figure1	4](#_Toc19731)**

**Supplemental [Table3	5](#_Toc32335)**

**Supplemental [Table4	6](#_Toc16246)**

**Supplemental [Table5	7](#_Toc30686)**

**Supplemental [Table6	9](#_Toc11199)**

**Supplemental [Table7	10](#_Toc18109)**

**Supplemental [Table8	11](#_Toc10260)**

**Supplemental [Table9	12](#_Toc10892)**

**Supplemental [Table10	13](#_Toc14169)**

# Table1 Associations of indoor and outdoor PM_2.5_ concentrations with dNLR in separate fixed-effects models

|  | Estimate | Std. Error | t value | *p* value |
| --- | --- | --- | --- | --- |
| Indoor PM_2.5_ | 0.221 | 0.083 | 2.681 | 0.008 |
| Outdoor PM_2.5_ | 0.031 | 0.015 | 2.104 | 0.036 |

Based on two waves of biomedical data from the Chinese Longitudinal Healthy Longevity Survey (CLHLS) collected in 2008 and 2014, we examined the associations between fine particulate matter (PM_2.5_) and inflammatory markers. Using individual fixed-effects models, We analyzed the associations of indoor and outdoor PM_2.5_ concentrations with the log-transformed derived neutrophil-to-lymphocyte ratio (dNLR), a marker of systemic inflammation. The dNLR was calculated as the difference between the total white blood cell count and lymphocyte count divided by the lymphocyte count and has been widely used as a marker of systemic inflammation. It has also been associated with the development and progression of several chronic diseases and mortality[1-3].Both indoor and outdoor PM_2.5_ concentrations were significantly and positively associated with dNLR (indoor PM_2.5_: $\beta$ = 0.221, SE = 0.083, P = 0.008; outdoor PM_2.5_: $\beta$ = 0.031, SE = 0.015, *p* = 0.036).

The fixed-effects analysis included 273 participants with repeated dNLR measurements, contributing 546 person-wave observations across the 2008 and 2014 survey waves. For each survey wave, indoor and outdoor PM_2.5_ exposures were assigned using the two-year average concentration for the survey year and the preceding year, consistent with the exposure assessment used in the main analysis. The fixed-effects models were adjusted using the same covariate specification as the fully adjusted models described in the main manuscript. These findings provide evidence of associations between PM_2.5_ exposure and dNLR but do not establish that PM_2.5_ causes systemic inflammation or that inflammation mediates the association between PM_2.5_ exposure and mortality. Because dNLR is a nonspecific marker of systemic inflammation, the results should be interpreted cautiously in view of potential residual confounding by other diseases, infections, medication use, and unmeasured environmental exposures.

Table2 Associations of single outdoor PM_2.5_ and its major components with mortality

|  |  | Partical_Model | Full_Model | |
| --- | --- | --- | --- | --- |
|  | HR | 95%CI | HR | 95%CI |
| PM_2.5_ | 1.001 | 1.000-1.003 | 1.002 | 1.000-1.003 |
| BC | 1.034 | 1.013-1.056 | 1.035 | 1.014-1.057 |
| OM | 1.003 | 0.999-1.007 | 1.004 | 1.000-1.008 |
| SO_4_^2-^ | 1.004 | 0.999-1.009 | 1.004 | 0.999-1.009 |
| NH_4_^+^ | 1.002 | 0.996-1.008 | 1.002 | 0.996-1.008 |
| NO_3_^-^ | 0.999 | 0.995-1.003 | 0.999 | 0.995-1.003 |

We explored the relationships between outdoor PM_2.5_ and its major components and mortality risk. Analyses of result showed that only BC was significantly associated with mortality risk. This finding differs from the indoor component analyses, in which indoor BC was most prominent but PM_2.5_, OM and SO_4_^2-^ also showed significant associations, whereas the outdoor BC–mortality relationship appeared to be more pronounced and PM_2.5_, OM and SO_4_^2-^ did not exhibit similar significance.

This discrepancy may be related to the higher dispersion of pollutants in outdoor environments. Outdoor PM_2.5_ tends to disperse rapidly in open spaces, where air movement dilutes many pollutants and may attenuate the detectable effects of some components on mortality. In contrast, BC is a highly fine and dense pollutant with strong stability and persistence in the atmosphere. It is often accompanied by higher concentrations of toxic co-pollutants, which may allow BC to remain airborne for longer periods and increase its potential for long-term health damage[4, 5].

Despite the mitigating role of outdoor air circulation on some pollutants, our findings underscore the particularly harmful role of BC in ambient pollution. BC has been linked to chronic respiratory diseases, cardiovascular diseases, and other adverse health outcomes, and older adults appear especially vulnerable to its effects[6-8]. Therefore, controlling outdoor BC concentrations and reducing combustion-related emissions are of great importance for improving population health and lowering mortality risk. Our results provide valuable evidence to guide policymakers in setting air quality control priorities.

Figure1 Spearman correlation coefficients among PM_2.5_ components

Note: iBC, indoor BC; iOM, indoor OM; iSO_4_^2-^, indoor SO_4_^2-^; iNH_4_+, indoor NH_4_+; iNO_3_^-^, indoor NO_3_^-^

Spearman correlation analyses indicated strong positive correlations among indoor PM_2.5_ components (correlation coefficients r = 0.75-0.98, all *p* < 0.001). The strongest correlation was observed between indoor NH_4_^+^ and NO_3_^-^ (r = 0.98), followed by indoor OM and SO_4_^2-^ (r = 0.96), and indoor BC and OM (r = 0.96), all showing very strong correlations. These findings suggest that, in our study population, different PM_2.5_ components may share common or similar pollution sources. Consequently, multicollinearity needs to be carefully addressed in subsequent multi-exposure models.

Table3 Multi-exposure models for indoor PM_2.5_ components and mortality risk

|  | **Partical_Model** |  | **Full_Model** | |
| --- | --- | --- | --- | --- |
|  | **HR** | **95%CI** | **HR** | **95%CI** |
| BC | 61.76 | 5.65，675.61 | 53.65 | 4.92，585.37 |
| OM | 1.40 | 1.07，1.83 | 1.38 | 1.05，1.81 |
| SO_4_^2-^ | 1.82 | 1.06，3.12 | 1.78 | 1.04，3.06 |
| NH_4_^+^ | 1.91 | 0.85，4.27 | 1.90 | 0.85，4.24 |
| NO_3_^-^ | 1.45 | 0.69，3.07 | 1.44 | 0.68，3.04 |

Building on the single-exposure analyses, we further conducted multi-exposure assessments to evaluate the associations between co-exposure to multiple indoor PM_2.5_ chemical components and all-cause mortality. Given the generally strong correlations among these components[9], directly including them simultaneously in the model would likely introduce substantial multicollinearity, thereby reducing the stability and interpretability of the effect estimates. To address this issue, we applied a regression-residual approach[10]. Specifically, each target pollutant was regressed on the other highly correlated components, and the linear portion explained by those components was removed. The remaining residuals—representing “de-correlated” exposure indicators—were then entered simultaneously into time-dependent Cox proportional hazards models to estimate the relatively independent effects of each PM_2.5_ component under indoor multi-exposure conditions.

In the indoor multi-exposure models, we constructed both partially adjusted and fully adjusted models to assess associations with all-cause mortality. The results showed that, indoor BC, OM, and SO_4_^2-^ consistently exhibited significant positive associations with all-cause mortality, which is consistent with the findings from the indoor single-exposure models.

These results highlight the distinct harmfulness of indoor BC, OM, and SO_4_^2-^. Even in scenarios where multiple indoor PM_2.5_ components coexist and exposure indicators “compete” to explain the outcome, these components retained independent and robust risk effects, suggesting that they may be key toxic constituents driving the adverse health impacts of indoor PM_2.5[11]_. Considering that older adults typically spend substantial amounts of time indoors and are particularly susceptible to indoor environmental influences, our findings underscore the need to prioritize the control of indoor BC emissions-primarily derived from combustion sources-while also addressing the contributions of OM and SO_4_^2-^. Strengthening indoor air quality management and implementing targeted emission reduction strategies may therefore be essential for effectively reducing mortality risk in older populations.

Table4 Multi-exposure models for outdoor PM_2.5_ components and mortality risk

|  | **Partical_Model** |  | **Full_Model** | |
| --- | --- | --- | --- | --- |
|  | **HR** | **95%CI** | **HR** | **95%CI** |
| BC | 6.88 | 1.58，29.95 | 6.40 | 1.47，27.79 |
| OM | 1.06 | 0.89，1.27 | 1.06 | 0.89，1.26 |
| SO_4_^2-^ | 1.19 | 0.85，1.66 | 1.18 | 0.84，1.65 |
| NH_4_^+^ | 0.92 | 0.54，1.56 | 0.92 | 0.54，1.58 |
| NO_3_^-^ | 0.72 | 0.43，1.21 | 0.72 | 0.43，1.21 |

We further applied multi-exposure models to outdoor PM_2.5_ components. Consistent with the indoor findings, both partially and fully adjusted models showed that only outdoor BC was significantly associated with all-cause mortality, while other outdoor PM_2.5_ constituents exhibited no statistically significant independent effects. This pattern is in line with the results from the single-pollutant outdoor analyses, indicating that BC consistently stands out as the most harmful component in both indoor and outdoor environments.

It is noteworthy that older adults spend most of their time indoors, where indoor BC may originate not only from outdoor infiltration but also from indoor combustion and cooking activities. Therefore, cumulative exposure to BC may be particularly relevant to their long-term health risks. Integrating the indoor and outdoor multi-exposure results suggests that BC is likely a key driver of the overall health effects of PM_2.5_. Our findings highlight the need to prioritize indoor BC exposure control, while simultaneously reducing outdoor combustion-related emissions, to effectively lower all-cause mortality risk among older adults.

Table5 Single indoor exposure with 1-year lag and mortality risk

|  | **Partical_Model** |  | **Full_Model** | |
| --- | --- | --- | --- | --- |
|  | **HR** | **95%CI** | **HR** | **95%CI** |
| BC | 1.0567 | 1.0214，1.0932 | 1.0580 | 1.0227，1.0944 |
| OM | 1.0056 | 0.9991，1.0122 | 1.0058 | 0.9992，1.0123 |
| SO_4_^2-^ | 1.0067 | 0.9984，1.0151 | 1.0068 | 0.9985，1.0152 |
| NH_4_^+^ | 1.0031 | 0.9931，1.0132 | 1.0029 | 0.9929，1.0131 |
| NO_3_^-^ | 0.9988 | 0.9926，1.0051 | 0.9987 | 0.9925，1.0050 |
| PM_2.5_ | 1.0024 | 1.0000，1.0048 | 1.0025 | 1.0000，1.0049 |

In our study, exposure assessment used a robust temporal window: the 2-year average indoor PM_2.5_ component concentrations in the year before death and the year of death were calculated as indicators of long-term exposure. This approach better captures cumulative exposure over a relatively long period and reduces the influence of short-term fluctuations on risk estimates. The main results showed that PM_2.5_, BC, OM, and SO_4_^2-^ were significantly associated with all-cause mortality, suggesting that these factors may affect older adults’ health under recent cumulative exposure conditions.

To test the robustness of these findings, we conducted sensitivity analyses focusing on lagged exposure, examining the associations between indoor PM_2.5_ and its major components with a 1-year lag and all-cause mortality. The results showed that only PM_2.5_ and BC remained significantly associated with mortality, whereas the associations of OM and SO_4_^2-^ were no longer statistically significant.

One possible explanation is that the 2-year average exposure window captures both short-term triggering effects and medium-term cumulative impacts, leading to detectable associations for OM and SO_4_^2-^. In contrast, the 1-year lag window emphasizes earlier exposure, and the persistence of the association for BC suggests a more sustained and robust health effect, while the effects of OM and SO_4_^2-^ may depend more on recent or episodic high exposures. Biologically, BC is known to induce chronic inflammation and oxidative stress over longer periods, which may explain its persistent significance in lagged analyses[12]. OM and sulfate, however, may act more through short-term inflammatory responses or in conjunction with transient environmental conditions, resulting in weaker effects when only earlier exposure is considered.

Overall, the main and sensitivity analyses consistently indicate that BC is the most robust long-term risk factor and should remain a primary target for indoor air quality interventions. At the same time, the potential short-term and triggering effects of OM and SO_4_^2-^ should not be overlooked and warrant attention in the management of acute exposure events and short-term pollution episodes.

Table6 Single outdoor exposure with 1-year lag and mortality risk

|  | **Partical_Model** |  | **Full_Model** | |
| --- | --- | --- | --- | --- |
|  | **HR** | **95%CI** | **HR** | **95%CI** |
| BC | 1.0342 | 1.0130，1.0558 | 1.0350 | 1.0138，1.0566 |
| OM | 1.0034 | 0.9994，1.0074 | 1.0035 | 0.9995，1.0075 |
| SO_4_^2-^ | 1.0041 | 0.9990，1.0092 | 1.0041 | 0.9991，1.0092 |
| NH_4_^+^ | 1.0019 | 0.9958，1.0081 | 1.0018 | 0.9957，1.0080 |
| NO_3_^-^ | 0.9993 | 0.9955，1.0031 | 0.9992 | 0.9954，1.0031 |
| PM_2.5_ | 1.0014 | 1.0000，1.0029 | 1.0015 | 1.0000，1.0030 |

Using the same 1-year lag structure as in the indoor analyses, we also evaluated single outdoor PM_2.5_ component exposures and mortality risk. Time-dependent Cox models were fitted separately for each component, adjusting for demographic characteristics, lifestyle factors, and baseline diseases. The results again showed that, in outdoor environments, only PM_2.5_ and BC was significantly and positively associated with all-cause mortality, while other factors did not show statistically significant effects.

These findings are highly consistent with the 1-year lagged indoor analyses: whether indoors or outdoors, BC exhibits more stable and persistent adverse health effects, suggesting that it is a key component driving increases in short- to medium-term mortality risk. Considering that older adults spend extended periods indoors and that outdoor BC can penetrate indoors, BC is likely to play a crucial role in shaping their health risks. This further supports the necessity and urgency of comprehensive control strategies targeting combustion-related BC emissions.

Table7 Multi-exposure models for indoor PM_2.5_ components with 1-year lag and mortality risk

|  | **Partical_Model** |  | **Full_Model** | |
| --- | --- | --- | --- | --- |
|  | **HR** | **95%CI** | **HR** | **95%CI** |
| BC | 14.88 | 1.15，192.36 | 12.08 | 0.99，164.89 |
| OM | 1.02 | 0.74，1.42 | 1.01 | 0.73，1.40 |
| SO_4_^2-^ | 1.15 | 0.70，1.92 | 1.14 | 0.68，1.89 |
| NH_4_^+^ | 0.70 | 0.32，1.52 | 0.71 | 0.33，1.53 |
| NO_3_^-^ | 0.52 | 0.26，1.04 | 0.52 | 0.26，1.05 |

In the 1-year lagged indoor multi-exposure sensitivity analyses, we simultaneously included residual-based exposure indicators for multiple indoor PM_2.5_ components in the same model to assess their independent associations with all-cause mortality under co-exposure conditions. After adjusting for demographic factors, lifestyle behaviors, and baseline chronic diseases, only indoor BC remained significantly and positively associated with mortality, while the other indoor components did not exhibit statistically significant effects in the lagged, multi-exposure context.

These results are consistent with the lagged single-exposure analyses and, from both the perspectives of multi-component co-exposure and temporal lag, further emphasize the robustness and dominant role of indoor BC in affecting health. On the one hand, in the multi-exposure models, BC must “compete” with other pollutants in explaining mortality risk yet still retains a significant association, suggesting that its effect is unlikely to be fully explained by co-occurring components or correlated exposures. On the other hand, the 1-year lag setting reflects the impact of earlier indoor cumulative exposure on subsequent mortality, and BC remains detectable and stable currently scale, supporting biologically plausible mechanisms involving chronic inflammation and long-term cardiopulmonary burden. Collectively, the 1-year lagged multi-exposure analyses further support the notion that reducing indoor BC exposure may be a key strategy to lower long-term mortality risk in older adults.

Table8 Multi-exposure models for outdoor PM_2.5_ components with 1-year lag and mortality risk

|  | **Partical_Model** |  | **Full_Model** | |
| --- | --- | --- | --- | --- |
|  | **HR** | **95%CI** | **HR** | **95%CI** |
| BC | 5.51 | 1.15，26.29 | 5.04 | 1.06，24.00 |
| OM | 1.03 | 0.85，1.26 | 1.03 | 0.84，1.25 |
| SO_4_^2-^ | 1.10 | 0.81，1.51 | 1.09 | 0.80，1.49 |
| NH_4_^+^ | 0.83 | 0.52，1.34 | 0.84 | 0.52，1.35 |
| NO_3_^-^ | 0.70 | 0.45，1.07 | 0.70 | 0.46，1.08 |

In the 1-year lagged outdoor multi-exposure models, when multiple outdoor PM_2.5_ components were entered simultaneously, only BC remained significantly and positively associated with all-cause mortality, and no independent effects were observed for other components. This pattern mirrors the indoor lagged analyses and further supports the conclusion that BC plays a robust and dominant role in long-term cumulative exposure effects on mortality among older adults. These findings underscore the critical importance of controlling BC emissions in long-term air quality management strategies.

Table9 The associations between indoor PM_2.5_ constituents and all‑cause mortality using an alternative infiltration factor of 0.41.

| **Model** | **Pollutant** | **HR(95%CI)** | ***p* value** |
| --- | --- | --- | --- |
| Full | BC | 1.1094(1.0522-1.1697) | 0.0001 |
| Full | OM | 1.0140(1.0037-1.0244) | 0.0076 |
| Full | SO_4_^2-^ | 1.0152(1.0022-1.0283) | 0.0216 |
| Full | NH_4_^+^ | 1.0133(0.9978-1.0289) | 0.0888 |
| Full | NO_3_^-^ | 1.0047(0.9953-1.0141) | 0.3288 |
| Full | PM_2.5_ | 1.0045(1.0011-1.0078) | 0.0087 |
| Partial | BC | 1.1118(1.0545-1.1721) | 0.0000 |
| Partial | OM | 1.0144(1.0041-1.0248) | 0.0061 |
| Partial | SO_4_^2-^ | 1.0154(1.0025-1.0285) | 0.0191 |
| Partial | NH_4_^+^ | 1.0134(0.9980-1.0289) | 0.0879 |
| Partial | NO_3_^-^ | 1.0047(0.9953-1.0142) | 0.3230 |
| Partial | PM_2.5_ | 1.0046(1.0012-1.0080) | 0.0071 |

Note: Partially adjusted models adjusted for age, sex, residence, marital status, education, smoking, alcohol, exercise, sleep quality, pension, BMI, and indoor temperature; fully adjusted models additionally adjusted for hypertension and diabetes.

Supplementary Table 9 presents the results of the sensitivity analysis using an alternative infiltration factor of 0.41 to estimate indoor pollutant concentrations, with the same Cox proportional hazards models as the main analysis. In both the fully adjusted and partially adjusted models, the associations remained robust and qualitatively unchanged[13]. Specifically, indoor BC, OM, SO₄²⁻, and PM₂.₅ were all significantly positively associated with all‑cause mortality, with hazard ratios of 1.109 (1.052–1.170), 1.014 (1.004–1.024), 1.015 (1.002–1.028), and 1.005 (1.001–1.008) per interquartile‑range increase in the full model, respectively; the partial model yielded nearly identical estimates. In contrast, NH₄⁺ and NO₃⁻ did not reach statistical significance (*p* = 0.088 and 0.329, respectively) in either model. These findings closely align with those obtained using the primary infiltration factor of 0.61 (main analysis), confirming that the observed associations are not sensitive to the choice of infiltration factor and supporting the reliability of our conclusions.

Table10 Subgroup analyses and interaction tests for the associations between outdoor PM_2.5_ components and all-cause mortality risk

| **Pollutant** | **Subgroup** | **Stratum** | **HR(95%CI)** | ***p* value** | ***p* for interaction** |
| --- | --- | --- | --- | --- | --- |
| BC | Residence | Urban | 1.009 (0.978-1.040) | 0.5906 | 0.0003 |
| BC | Residence | Rural | 1.074 (1.041-1.108) | 0 | 0.0003 |
| BC | Sex | Male | 1.023 (0.989-1.058) | 0.1862 | 0.084 |
| BC | Sex | Female | 1.059 (1.029-1.089) | 0.0001 | 0.084 |
| BC | Age | ≤90 | 1.033 (0.996-1.072) | 0.0793 | 0.0114 |
| BC | Age | 91-100 | 1.029 (0.994-1.064) | 0.102 | 0.0114 |
| BC | Age | >100 | 1.093 (1.045-1.144) | 0.0001 | 0.0114 |
| OM | Residence | Urban | 1.000 (0.994-1.006) | 0.9845 | 0.0029 |
| OM | Residence | Rural | 1.010 (1.004-1.015) | 0.001 | 0.0029 |
| OM | Sex | Male | 1.003 (0.997-1.010) | 0.3042 | 0.2427 |
| OM | Sex | Female | 1.007 (1.002-1.013) | 0.0086 | 0.2427 |
| OM | Age | ≤90 | 1.007 (1.000-1.015) | 0.0427 | 0.1757 |
| OM | Age | 91-100 | 1.002 (0.996-1.009) | 0.4771 | 0.1757 |
| OM | Age | >100 | 1.011 (1.003-1.020) | 0.0095 | 0.1757 |
| NH_4_^+^ | Residence | Urban | 0.999 (0.988-1.009) | 0.8095 | 0.0672 |
| NH_4_^+^ | Residence | Rural | 1.008 (1.000-1.016) | 0.06 | 0.0672 |
| NH_4_^+^ | Sex | Male | 1.003 (0.993-1.013) | 0.5084 | 0.3255 |
| NH_4_^+^ | Sex | Female | 1.007 (0.999-1.015) | 0.0982 | 0.3255 |
| NH_4_^+^ | Age | ≤90 | 1.008 (0.997-1.019) | 0.151 | 0.4677 |
| NH_4_^+^ | Age | 91-100 | 1.003 (0.993-1.013) | 0.6148 | 0.4677 |
| NH_4_^+^ | Age | >100 | 1.012 (0.999-1.025) | 0.0672 | 0.4677 |
| NO_3_^-^ | Residence | Urban | 0.997 (0.991-1.004) | 0.4038 | 0.0461 |
| NO_3_^-^ | Residence | Rural | 1.003 (0.999-1.008) | 0.1575 | 0.0461 |
| NO_3_^-^ | Sex | Male | 1.001 (0.995-1.007) | 0.7057 | 0.4712 |
| NO_3_^-^ | Sex | Female | 1.002 (0.997-1.007) | 0.335 | 0.4712 |
| NO_3_^-^ | Age | ≤90 | 1.005 (0.998-1.011) | 0.1775 | 0.4774 |
| NO_3_^-^ | Age | 91-100 | 1.000 (0.994-1.006) | 0.9445 | 0.4774 |
| NO_3_^-^ | Age | >100 | 1.004 (0.997-1.012) | 0.2652 | 0.4774 |
| SO_4_^2-^ | Residence | Urban | 0.999 (0.991-1.007) | 0.8186 | 0.0067 |
| SO_4_^2-^ | Residence | Rural | 1.010 (1.003-1.017) | 0.0047 | 0.0067 |
| SO_4_^2-^ | Sex | Male | 1.003 (0.995-1.012) | 0.4294 | 0.221 |
| SO_4_^2-^ | Sex | Female | 1.008 (1.001-1.015) | 0.0194 | 0.221 |
| SO_4_^2-^ | Age | ≤90 | 1.006 (0.997-1.015) | 0.2083 | 0.1227 |
| SO_4_^2-^ | Age | 91-100 | 1.004 (0.996-1.012) | 0.3606 | 0.1227 |
| SO_4_^2-^ | Age | >100 | 1.013 (1.003-1.024) | 0.0144 | 0.1227 |
| PM_2.5_ | Residence | Urban | 1.000 (0.998-1.002) | 0.9166 | 0.0076 |
| PM_2.5_ | Residence | Rural | 1.003 (1.001-1.005) | 0.0013 | 0.0076 |
| PM_2.5_ | Sex | Male | 1.002 (1.000-1.004) | 0.1246 | 0.6537 |
| PM_2.5_ | Sex | Female | 1.002 (1.000-1.004) | 0.0319 | 0.6537 |
| PM_2.5_ | Age | ≤90 | 1.003 (1.001-1.006) | 0.0044 | 0.1775 |
| PM_2.5_ | Age | 91-100 | 1.001 (0.999-1.003) | 0.3505 | 0.1775 |
| PM_2.5_ | Age | >100 | 1.002 (1.000-1.005) | 0.0844 | 0.1775 |

We further examined the associations of outdoor PM₂.₅ and its components with all‑cause mortality across strata of residence, sex, and age, and formally tested for effect modification via interaction terms.

**Reference**

1.	Mezquita L, Auclin E, Ferrara R, Charrier M, Remon J, Planchard D, Ponce S, Ares LP, Leroy L, Audigier-Valette C *et al*: Association of the Lung Immune Prognostic Index With Immune Checkpoint Inhibitor Outcomes in Patients With Advanced Non-Small Cell Lung Cancer. *JAMA oncology* 2018, 4(3):351-357.https://doi.or/10.1001/JamaonCol.2017.4771

2.	Zhang J, Fan X, Xu Y, Wang K, Xu T, Han T, Hu C, Li R, Lin X, Jin L: Association between inflammatory biomarkers and mortality in individuals with type 2 diabetes: NHANES 2005-2018. *Diabetes research and clinical practice* 2024, 209:111575.https://doi.or/10.1016/J.diabres.2024.111575

3.	Guo B, Liu X, Si Q, Zhang D, Li M, Li X, Zhao Y, Hu F, Zhang M, Liu Y *et al*: Associations of CBC-Derived inflammatory indicators with sarcopenia and mortality in adults: evidence from Nhanes 1999 ∼ 2006. *BMC geriatrics* 2024, 24(1):432.https://doi.or/10.1186/s12877-024-05012-2

4.	Janssen NA, Hoek G, Simic-Lawson M, Fischer P, van Bree L, ten Brink H, Keuken M, Atkinson RW, Anderson HR, Brunekreef B *et al*: Black carbon as an additional indicator of the adverse health effects of airborne particles compared with PM10 and PM2.5. *Environmental health perspectives* 2011, 119(12):1691-1699.https://doi.or/10.1289/ehp.1003369

5.	Zhu A, Kan H, Shi X, Zeng Y, Ji JS: Black Carbon Air Pollution and Incident Mortality Among the Advance-Aged Adults in China: A Prospective Cohort Study. *The journals of gerontology Series A, Biological sciences and medical sciences* 2025, 80(4).doi: https://doi.or/10.1093/gerona/glae302

6.	Liu S, Geng G, Xiao Q, Zheng Y, Liu X, Cheng J, Zhang Q: Tracking Daily Concentrations of PM(2.5) Chemical Composition in China since 2000. *Environmental science & technology* 2022, 56(22):16517-16527.https://doi.or/10.1021/acs.est.2c06510

7.	Cui C, Liu Y, Chen L, Liang S, Shan M, Zhao J, Liu Y, Yu S, Sun Y, Mao J *et al*: Assessing public health and economic loss associated with black carbon exposure using monitoring and MERRA-2 data. *Environmental pollution (Barking, Essex : 1987)* 2022, 313:120190.https://doi.or/10.1016/j.envpol.2022.120190

8.	Qi J, Zhao N, Liu M, Guo Y, Fu J, Zhang Y, Wang W, Su Z, Zeng Y, Yao Y *et al*: Long-term exposure to fine particulate matter constituents and cognitive impairment among older adults: An 18-year Chinese nationwide cohort study. *J Hazard Mater* 2024, 468:133785.https://doi.or/10.1016/j.jhazmat.2024.133785

9.	Zhu G, Wen Y, Du R, Cao K, Zhang R, Lu X, Liang J, Gao Q, Wang T: Sustainable development reduces particulate matter emissions and mitigates aging’s cognitive impact. *npj Climate and Atmospheric Science* 2025, 8(1):176.https://doi.org/10.1038/s41612-025-01052-6

10.	Yang BY, Guo Y, Markevych I, Qian ZM, Bloom MS, Heinrich J, Dharmage SC, Rolling CA, Jordan SS, Komppula M *et al*: Association of Long-term Exposure to Ambient Air Pollutants With Risk Factors for Cardiovascular Disease in China. *JAMA network open* 2019, 2(3):e190318.https://doi.org/10.1001/jamanetworkopen.2019.0318

11.	Shi Y, Wu D, Yilihamu Y, Zheng Y, Zhang L: Impact of long-term exposure to PM(2.5) and its components on the risk of chronic lung disease in middle-aged and elderly chinese: evidence from a large Chinese population-based cohort. *BMC Public Health* 2025, 25(1):3301.https://doi.org/ 10.1186/s12889-025-24704-x

12.	Holz O, Heusser K, Müller M, Windt H, Schwarz K, Schindler C, Tank J, Hohlfeld JM, Jordan J: Airway and systemic inflammatory responses to ultrafine carbon black particles and ozone in older healthy subjects. *Journal of toxicology and environmental health Part A* 2018, 81(13):576-588.https://doi.org/10.1080/15287394.2018.1463331

13.	Shi S, Chen C, Zhao B: Modifications of exposure to ambient particulate matter: Tackling bias in using ambient concentration as surrogate with particle infiltration factor and ambient exposure factor. *Environmental pollution (Barking, Essex : 1987)* 2017, 220(Pt A):337-347.<https://doi.org/10.1016/j.envpol.2016.09.069>
